# Xyloketal B Attenuates Atherosclerotic Plaque Formation and Endothelial Dysfunction in Apolipoprotein E Deficient Mice

**DOI:** 10.3390/md13042306

**Published:** 2015-04-14

**Authors:** Li-Yan Zhao, Jie Li, Feng Yuan, Mei Li, Quan Zhang, Yun-Ying Huang, Ji-Yan Pang, Bin Zhang, Fang-Yun Sun, Hong-Shuo Sun, Qian Li, Lu Cao, Yu Xie, Yong-Cheng Lin, Jie Liu, Hong-Mei Tan, Guan-Lei Wang

**Affiliations:** 1 Department of Pharmacology, Zhongshan School of Medicine, Sun Yat-sen University, Guangzhou 510080, China; E-Mails: yuwuwei@live.com (L.-Y.Z.); yuanfeng@mail2.sysu.edu.cn (F.Y.); 290496167@qq.com (Y.X.); liujie@mail.sysu.edu.cn (J.L.); 2 Department of Anesthesiology, Sun Yat-sen Memorial Hospital, Sun Yat-sen University, Guangzhou 510080, China; E-Mail: mdlijie@sina.com; 3 VIP Healthcare Center, The Third Affiliated Hospital of Sun Yat-sen University, Guangzhou 510630, China; E-Mail: limeimed@163.com; 4 Department of Pathophysiology, Zhongshan School of Medicine, Sun Yat-sen University, Guangzhou 510080, China; E-Mails: 543450713@qq.com (Q.Z.); 2558418023@qq.com (Q.L.); caolu11192@126.com (L.C.); 5 Department of Pharmacy, The fifth Affiliated Hospital of Guangzhou Medical University, Guangzhou 510182, China; E-Mail: remember917@163.com; 6 Department of Applied Chemistry, School of Chemistry and Chemical Engineering, Sun Yat-sen University, Guangzhou 510080, China; E-Mails: cespjy@mail.sysu.edu.cn (J.-Y.P.); ceslyc@mail.sysu.edu.cn (Y.-C.L.); 7 Department of Education of Guangdong Province, Guangdong Province Key Laboratory of Functional Molecules in Oceanic Microorganism, Sun Yat-sen University, Guangzhou 510080, China; 8 Guangdong Cardiovascular Institute, Guangdong General Hospital, Guangzhou 510080, China; E-Mail: drbinzhang@163.com; 9 Lab for Basic Research of Life Science, School of Medicine, Tibet Institute for Nationalities, Xianyang 712082, China; E-Mails: xzmysfy@163.com; 10 Departments of Surgery and Physiology, Institute of Medical Science, Faculty of Medicine, University of Toronto, Toronto, ON M5G 1G6, Canada; E-Mail: hss.sun@utoronto.ca

**Keywords:** xyloketals, atherosclerosis, endothelium, eNOS, apoE-deficient mice

## Abstract

Our previous studies demonstrated that xyloketal B, a novel marine compound with a unique chemical structure, has strong antioxidant actions and can protect against endothelial injury in different cell types cultured *in vitro* and model organisms *in vivo*. The oxidative endothelial dysfunction and decrease in nitric oxide (NO) bioavailability are critical for the development of atherosclerotic lesion. We thus examined whether xyloketal B had an influence on the atherosclerotic plaque area in apolipoprotein E-deficient (apoE^−/−^) mice fed a high-fat diet and investigated the underlying mechanisms. We found in our present study that the administration of xyloketal B dose-dependently decreased the atherosclerotic plaque area both in the aortic sinus and throughout the aorta in apoE^−/−^ mice fed a high-fat diet. In addition, xyloketal B markedly reduced the levels of vascular oxidative stress, as well as improving the impaired endothelium integrity and NO-dependent aortic vasorelaxation in atherosclerotic mice. Moreover, xyloketal B significantly changed the phosphorylation levels of endothelial nitric oxide synthase (eNOS) and Akt without altering the expression of total eNOS and Akt in cultured human umbilical vein endothelial cells (HUVECs). Here, it increased eNOS phosphorylation at the positive regulatory site of Ser-1177, while inhibiting phosphorylation at the negative regulatory site of Thr-495. Taken together, these findings indicate that xyloketal B has dramatic anti-atherosclerotic effects *in vivo*, which is partly due to its antioxidant features and/or improvement of endothelial function.

## 1. Introduction

Atherosclerosis (AS) is currently the leading cause of death among the cardiovascular diseases. Endothelial dysfunction is an early key event during the atherogenesis [1] and has been identified as a common link of all cardiovascular risk factors, including dyslipidemia [2], hypertension [3], hyperhomocysteinemia [4], diabetes [5], and smoking [6], in the vascular system. Two basic functions of the endothelium associated with lesion development in athero-susceptible regions include maintenance of the vascular permeability barrier and guarantee of nitric oxide (NO) bioavailability [7]. Loss of NO bioavailability is majorly due to reduced synthesis of NO and increased reactive oxygen species (ROS) accumulation, and is a cardinal feature of endothelial dysfunction during the development of atherosclerosis [8]. NO exerts many protective effects in the cardiovascular system, such as decrease of blood pressure, regulation of vascular tone, promotion of endothelial cell proliferation, inhibition of platelet aggregation and leukocyte adhesion, and prevention of smooth muscle cell proliferation [9]. Endothelial nitric oxide synthase (eNOS) is the enzyme responsible for NO generation in endothelial cells and is the primary physiological source of NO [10]. Due to endothelial NO properties, eNOS is therefore taken as an important endogenous anti-atherogenic mechanism and also serves as a potential drug target for the prevention or treatment of AS. Also, several phosphorylation sites within eNOS have been identified as regulating eNOS activity [11]. Compounds that increase NO bioavailability and eNOS activity are of therapeutic interest.

Xyloketal B is a novel marine compound with a unique chemical structure [12] isolated from mangrove fungus Xylaria sp. (no. 2508) [13,14]. The accumulating evidence has well established that xyloketal B has strong antioxidant actions in several model systems [15,16,17,18,19]. Xyloketal B can directly scavenge 1,1-diphenyl-2-picrylhydrazyl (DPPH) free radical production [17] and can attenuate oxidized low density lipoprotein (oxLDL)-induced ROS generation [15]. Recently, we found that xyloketal B exhibits its antioxidant activity through induction of HO-1 in vascular endothelial cells and zebrafish [18]. In human umbilical vein endothelial cells (HUVECs), xyloketal B alone could induce NO generation, and peroxinitrate formation in parallel with protecting against oxLDL-induced cell injury. These findings indicate that xyloketal B is an effective antioxidant and may preserve NO bioavailability in the presence of elevated ROS. Furthermore, the aortic tension experiments showed that xyloketal B significantly improves endothelium-dependent, NO-mediated vasorelaxation to acetylcholine (Ach); whereas this treatment effect was eliminated in the presence of eNOS blocker *N*-nitro-l-arginine methyl ester (l-NAME), suggesting that xyloketal B may improve endothelial dysfunction through enhancing NO bioavailability [16].

Oxidative endothelial injury and decrease in NO bioavailability are critical for atherosclerotic lesion formation, which have therefore been the drug targets for the treatment of atherosclerosis. However, new drugs targeting on protecting vascular endothelium and against oxidative stress are still lacking for clinical use. Given that xyloketal B is very safe and a strong anti-oxidant with protective effects against endothelial cell injury *in vitro*, we hypothesized that xyloketal B has anti-atherosclerotic effects *in vivo* by reducing oxidative stress and improving endothelial dysfunction. In the present study, we initially explored whether xyloketal B could attenuate atherosclerotic plaque area both in the aortic sinus and throughout the aorta from apolipoprotein E-deficient (apoE^−/−^) mice fed a high-fat diet. In atherosclerotic apoE^−/−^ mice, we then investigated the mechanism of the anti-atherosclerotic effects of xyloketal B by reducing a high degree of oxidative stress and improving impaired endothelial dysfunction. We also investigated the effects of xyloketal B on eNOS phosphorylation in cultured HUVECs. Our present study indicates that xyloketal B has a dramatic anti-atherosclerotic effect *in vivo*, which is partly due to its antioxidant features and/or improvement of endothelial function.

## 2. Results

### 2.1. Effect of Xyloketal B on High-Fat Diet Induced Atherosclerotic Lesion Formation in ApoE^−/−^ Mice

**Figure 1:**
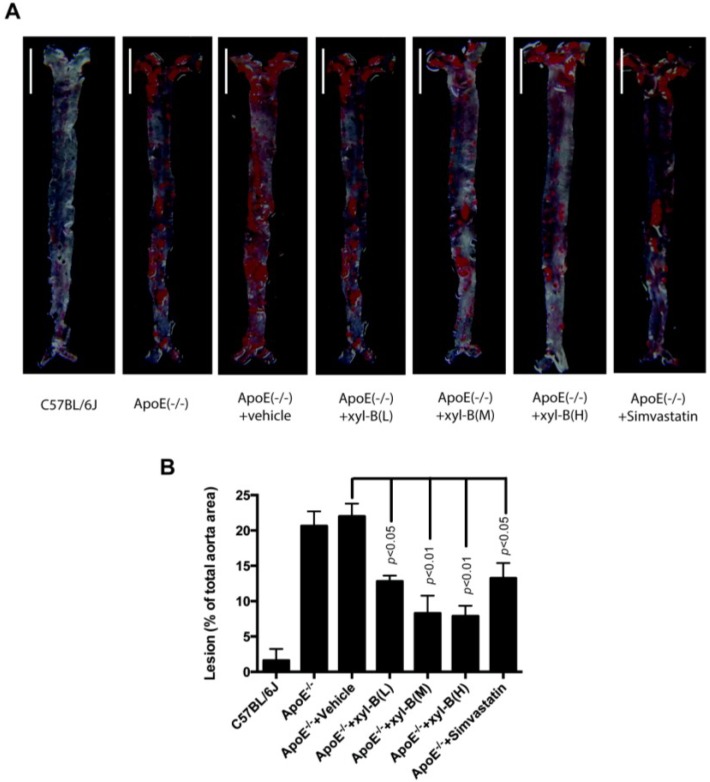
Effect of xyloketal B treatment on atherosclerotic lesions in aortas of apoE^−/−^ mice fed a high fat diet. ApoE^−/−^ mice were fed with high-fat diet with or without xyloketal B/simvastatin treatment for 16 weeks, C57BL/6J mice fed with high-fat diet served as control. (**A**) The aortic atherosclerotic lesions in C57BL/6J, ApoE(−/−), ApoE(−/−) + vehicle, ApoE(−/−) + xyl-B (L, M, H) and ApoE(−/−) + Simvastatin group were examined by en face oil red O staining in aortas, and the representative image of each group is shown, scale bar = 0.5 cm; (**B**) Lesions were expressed as the percentage of positive staining for oil-red O of total aortic area (%). Significant differences are shown in the bar graph by *p* < 0.01 or *p* < 0.05 *vs.* ApoE^−/−^ + vehicle, *n* = 8 mice. ApoE^−/−^ + xyl-B (L, M, H): ApoE^−/−^ mice treated with 7 mg/kg/day, 14 mg/kg/day or 28 mg/kg/day of xyloketal B.

To investigate the influence of xyloketal B on atherosclerosis, we fed apoE^−/−^ mice with high-fat diet with or without xyloketal B/simvastatin treatment for 16 weeks, and then evaluated the atherosclerotic lesion area in the aortic arch, thoracic and abdominal aorta. The en face oil-red O staining of aorta was performed to determine the lesion area covering the aortic surface. As shown in Figure 1, there is no obvious lesion in the aorta and aortic sinus of C57BL/6J mice fed with high-fat diet. In all apoE^−/−^ mice, however, we observed apparent atherosclerotic oil-red O staining in the aortic arch, thoracic, and abdominal aorta, which could be significantly attenuated by xyloketal B and simvastatin. In apoE^−/−^ mice/vehicle-treated apoE^−/−^ mice, approximately 20.6%/21.9% of the aortic surface was covered by atherosclerotic plaques, and there was no difference between these two groups. The positive staining percentages in xyloketal B treated mice were 12.8%, 8.3%, and 7.9% at the low dose (L, 7 mg/kg/day), medium dose (M, 14 mg/kg/day) and high dose (H, 28 mg/kg/day), respectively. It was 13.6% in simvastatin treated mice at a dose of 10 mg/kg/day. The groups of xyloketal B at medium and high doses could reduce the atherosclerotic lesion area by nearly two-thirds in comparison to the vehicle-treated apoE^−/−^ group, indicating its stronger influence on atherosclerotic progression than simvastatin. The data indicated that treatment of xyloketal B could reduce the aortic atherosclerotic lesion area in the entire aorta in a concentration dependent manner.

**Figure 2:**
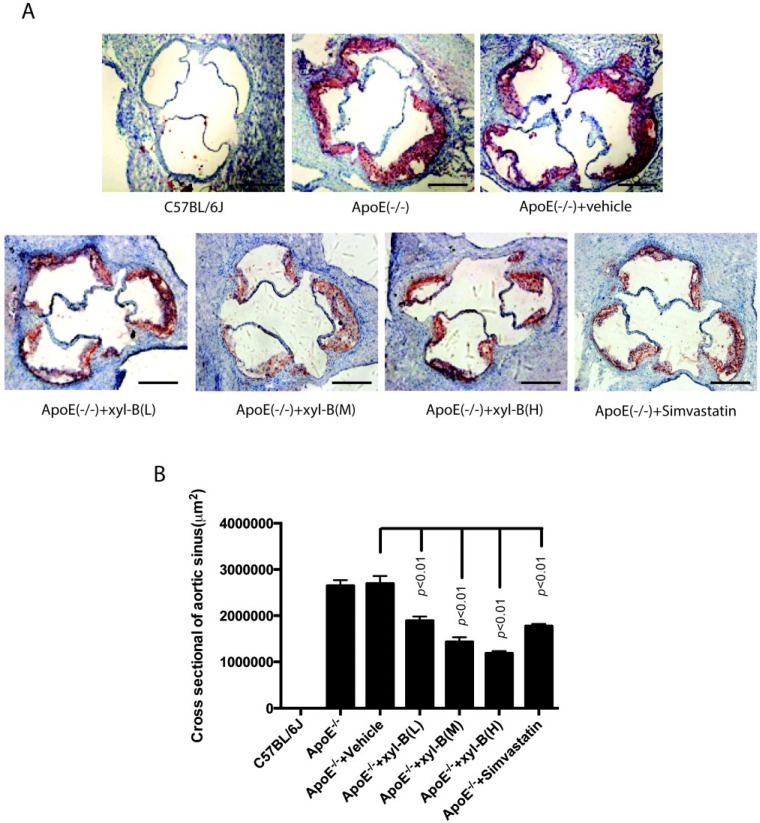
Effect of xyloketal B treatment on atherosclerotic lesions in the aortic sinus in apoE^−/−^ mice. ApoE^−/−^ mice were fed with high-fat diet with or without xyloketal B/simvastatin treatment for 16 weeks, C57BL/6J mice fed with high-fat diet served as control. (**A**) The atherosclerotic plaques in the aortic sinus in C57BL/6J, ApoE(−/−), ApoE(−/−) + vehicle, ApoE(−/−) + xyl-B (L, M, H) and ApoE(−/−) + Simvastatin group were examined by oil red O staining and representative image of each group is shown, scale bar = 400 μm; (**B**) The area of positive staining for oil-red O (μm^2^) was calculated. Significant differences are shown in the bar graph by *p* < 0.01, *n* = 8 mice. ApoE^−/−^ + xyl-B (L, M, H): ApoE^−/−^ mice treated with 7 mg/kg/day, 14 mg/kg/day or 28 mg/kg/day of xyloketal B.

As shown in Figure 2, we got consistent results in the aortic sinus using cross-sectional analysis. After 16 weeks of high-fat diet, atherosclerotic plaques in the aortic sinus were obvious in apoE^−/−^ mice (Figure 2A) and the plaque areas were significantly reduced by the treatment of xyloketal B or simvastatin. At the dose of 7, 14, or 28 mg/kg/day, xyloketal B induced a reduction in plaque areas of 803,000 μm^2^, 1,262,000 μm^2^ and 1,508,000 μm^2^, respectively, in comparison to the corresponding vehicle-treated apoE^−/−^ mice (Figure 2B).

Meanwhile, as shown in Table 1, xyloketal B had no effect on body weight in atherosclerotic apoE^−/−^ mice. In another parallel study, we also found that xyloketal B had beneficial effects on lipid metabolism disorder, such as decreasing plasma total cholesterol (TC) [20]. These findings were consistent with the effect of simvastatin.

**Table 1:** Body weight of mice (g). No significant difference was observed among all groups at 0, 8, 16 weeks, respectively. Data are shown as mean ± SEM. *n* = 8.

	C57BL/6J	ApoE^−/−^	ApoE^−/−^ + Vehicle	ApoE^−/−^ + xyl-B (M, 14 mg/kg/day)	ApoE^−/−^ + Simvastatin (10mg/kg/day)
0 week	18 ± 0.26	18 ± 0.32	19 ± 0.42	18 ± 0.36	18 ± 0.28
8 weeks	24 ± 0.53	26 ± 0.52	25 ± 0.49	24 ± 0.61	24 ± 0.41
16 weeks	29 ± 0.62	30 ± 0.77	31 ± 0.59	30 ± 0.69	30 ± 0.53

### 2.2. Effect of Xyloketal B on the Level of Serum Autoantibodies against oxLDL in Atherosclerotic Apoe^−/−^ Mice

**Figure 3:**
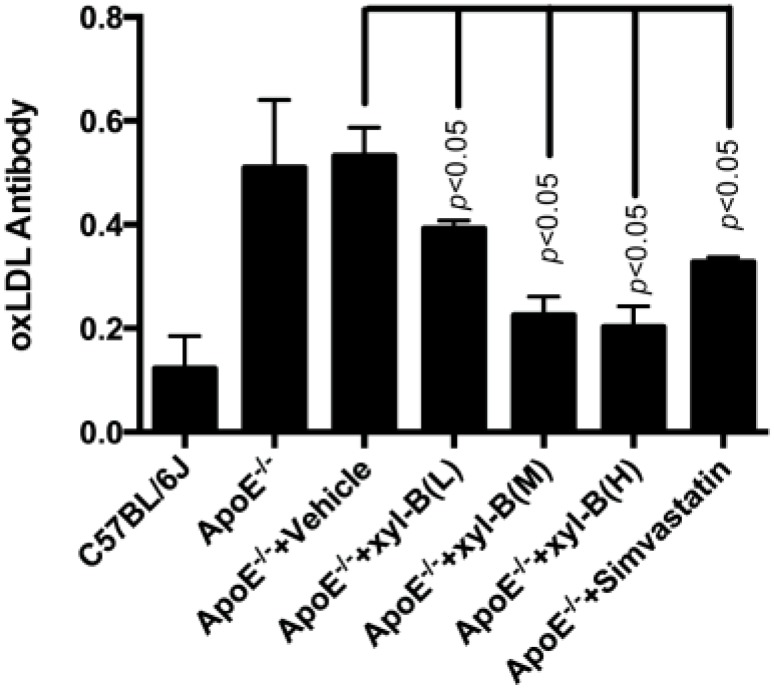
Effect of xyloketal B on the level of serum autoantibody against oxLDL in apoE^−/−^ mice. ApoE^−/−^ mice were fed with high-fat diet with or without xyloketal B/simvastatin treatment for 16 weeks, C57BL/6J mice fed with high-fat diet served as control. Serum was obtained from C57BL/6J, ApoE^−/−^, ApoE^−/−^ + Vehicle, ApoE^−/−^ + xyl-B (L, M, H) and ApoE^−/−^ + Simvastatin group, the serum titer of anti-oxidized low-density lipoprotein (oxLDL) autoantibodies was measured by an enzyme-linked immunosorbent assay. Significant differences are shown in the bar graph by *p* < 0.05, *n* = 8. ApoE^−/−^ + xyl-B (L, M, H): ApoE^−/−^ mice treated with 7 mg/kg/day, 14 mg/kg/day or 28 mg/kg/day of xyloketal B.

Because xyloketal B has been shown to have strong antioxidant actions in different *in vitro* cellular models and *in vivo* model organisms [16], here we examined whether it had *in vivo* antioxidant actions against atherosclerosis. As shown in Figure 3, there was a significant increase in the serum anti-oxLDL antibody titer in apoE^−/−^ mice compared with C57BL/6J mice (both fed with high fat diet). Compared with corresponding vehicle-treated apoE^−/−^ mice, administration of xyloketal B at 7, 14, 28 mg/kg/day significantly reduced the titers of serum oxLDL antibody. Xyloketal B at a dose of 7 mg/kg/day could reduce the serum oxLDL antibody level by about 30%, which is comparable to simvastatin (35% at 10 mg/kg/day). Additionally xyloketal B exhibited stronger antioxidant actions at higher doses, which might contribute to preventive effects of xyloketal B against atherosclerosis.

### 2.3. Protective Effects of Xyloketal B on Endothelial Integrity during Atherosclerosis

**Figure 4:**
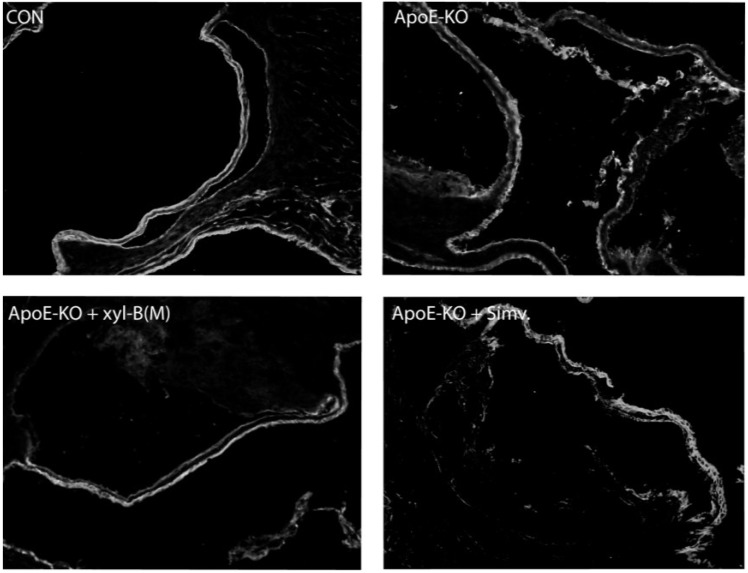
Effect of xyloketal B on endothelial integrity in aortic sinus from apoE^−/−^ mice. ApoE^−/−^ mice fed with high-fat diet were treated with vehicle or xyloketal B (14 mg/kg/day) or simvastatin (10 mg/kg/day) for 8 weeks, respectively, and C57BL/6J mice fed with high-fat diet served as control. Immunofluorescence staining of PECAM-1, a marker of endothelium, was performed in the aortic sinus from CON, ApoE-KO, ApoE-KO + Xyl-B(M) and ApoE-KO + Simv. group, Representative sections were randomly chosen from at least 6 mice for each group, and are shown by fluorescence confocal microscopy (200×).

**Figure 5:**
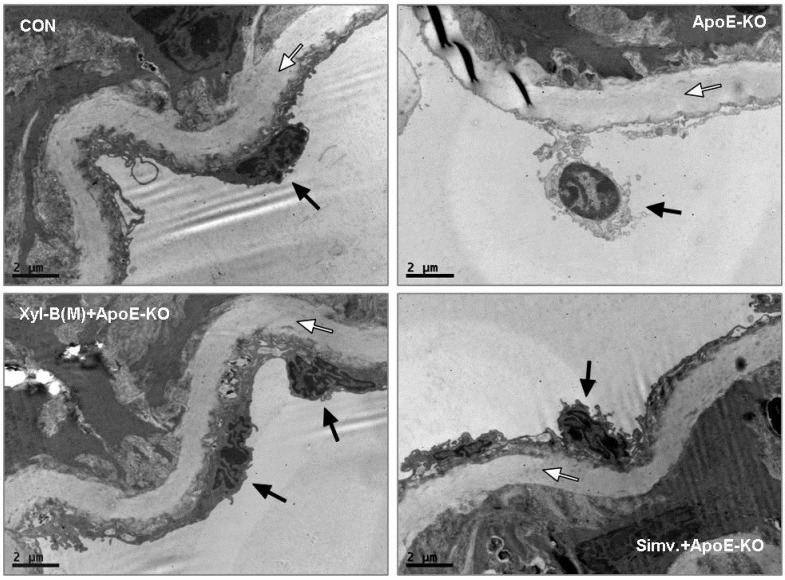
Effect of xyloketal B on endothelial integrity in thoracic aortas in apoE^−/−^ mice. ApoE^−/−^ mice fed with high-fat diet were treated with vehicle or xyloketal B (14 mg/kg/day) or simvastatin (10 mg/kg/day) for 8 weeks, respectively, C57BL/6J mice fed with high-fat diet served as control. Micrographs of aortic sections were captured using transmission electron. Internal elastic lamina was observed clearly (white arrows) in the aortas of all mice. Endothelial layer was next to the internal elastic lamina (between the internal elastic lamina and the lumen). The vascular wall of the control group was characterized by a continuous layer of endothelial cells (ECs) while EC detachment (black arrow) was observed in the aortas from ApoE-KO group. Aortas from ApoE-KO + Xyl-B(M) and ApoE-KO + Simv. groups exhibited a continuous layer of EC (black arrows) with no alteration compared with control C57BL/6J mice. Bar = 2 μm.

To examine whether xyloketal B administration can improve endothelial integrity *in vivo* during atherosclerosis, apoE^−/−^ mice fed with high-fat diet were treated with vehicle or xyloketal B (14 mg/kg/day) or simvastatin (10 mg/kg/day), respectively, for eight weeks, C57BL/6J mice fed with high-fat diet served as control group. Immunofluorescence staining with antibody against PECAM-1 was performed in the aortic sinus sections from different groups. PECAM-1, which is abundantly expressed on the surfaces of many cells including monocytes, lymphocytes, platelets, and endothelial cells [7], is a sensitive and specific marker of endothelium. In Figure 4, the representative fluorescent images showed that PECAM-1 was expressed predominantly in the endothelium coating the internal surface of aortic valves, and indicated how endothelium integrity changes during the atherosclerosis. The loss of endothelial integrity was clearly defined in the apoE^−/−^-model group, appearing as an intermittent line of PECAM-1 fluorescence in endothelium, which could be significantly improved by the treatment of xyloketal B or simvastatin. We also detected the ultrastructural changes of endothelial cells (ECs) in aortic vessels from different groups using transmission electron microscopic experiments. As shown in Figure 5, ECs of aortas from C57BL/6J mice exerted normal ultrastructure, while the EC detachment was observed in apoE^−/−^ mice. Compared with apoE^−/−^ mice, xyloketal B- or simvastatin-treated group exhibited a relatively normal appearance of endothelium. These results indicated that xyloketal B could maintain vascular endothelial integrity against atherosclerosis.

### 2.4. Xyloketal B Improved NO-Dependent Aortic Vasorelaxation in Atherosclerotic Apoe^−/−^ Mice

**Figure 6:**
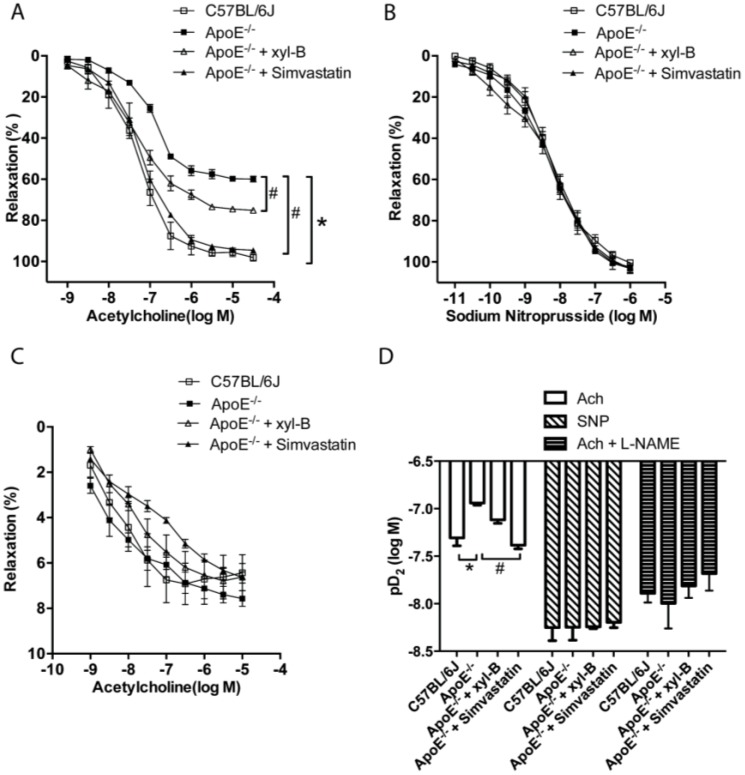
Effect of xyloketal B treatment on relaxation of aortic rings isolated from apoE^−/−^ mice. ApoE^−/−^ mice fed with high-fat diet were treated with vehicle or xyloketal B (14 mg/kg/day) or simvastatin (10 mg/kg/day), respectively, for 16 weeks, C57BL/6J mice fed with high-fat diet served as control. Tension of aorta rings from C57BL/6J, ApoE^−/−^, ApoE^−/−^ + xyl-B and ApoE^−/−^ + simvastatin groups were measured with a myograph system. Aortic rings were pre-contracted with phenylephrine (10^−7^ M) and then (**A**) acetylcholine (Ach, 10^−9^ to 3 × 10^−5^ M) or (**B**) sodium nitroprusside (SNP, 10^−11^ to 10^−6^ M) was added to induce relaxation; (**C**) Aortic rings were pre-incubated for 20 min with 30 μM l-NAME and pre-contracted with phenylephrine (10^−7^ M), then Ach (10^−9^ to 3 × 10^−5^ M) induced relaxation were recorded. Concentration-response curves are shown in (A), (B) and (C), vasorelaxation is expressed as the reduction percentage of the total vascular tone; (**D**) Half-maximal response dose (expressed as pD_2_) of Ach, SNP and Ach + l-NAME were calculated. * *p* < 0.05 *vs.*C57BL/6J group, # *p* < 0.05 *vs.* ApoE^−/−^ group, *n* = 4~6.

Our previous studies demonstrated that xyloketal B and other xyloketal derivatives can relax rat isolated thoracic aorta and promote endothelial NO production [16]. We therefore further investigated the effect of xyloketal B on endothelium/NO dependent [acetylcholine (Ach)-induced], endothelium-derived NO independent [sodium nitroprusside (SNP)-induced] and NO independent (NOS inhibitor l-NAME pretreated, Ach-induced) vasorelaxation activity in thoracic aorta from the atherosclerotic model. The arterial tension measurement of the thoracic aorta was performed as we previously described [16], and Ach-induced vasorelaxation was measured in the aorta rings that were pre-contracted by phenylephrine. As shown in Figure 6A, Ach-induced maximal response was significantly reduced in apoE^−/−^ group (ApoE^−/−^ 60% ± 2% *vs.* C57BL/6J 98% ± 3% at 3 × 10^−5^ moL/L Ach, *p* < 0.05). These data indicated that endothelium/NO dependent vasorelaxation was significantly impaired in apoE^−/−^ mice. Both xyloketal B (14 mg/kg/day) and simvastatin (10 mg/kg/day) treatment could improve the impaired vasorelaxation by shifting the relaxation curve to the left and increasing the maximal response (ApoE^−/−^ + xyl-B 75% ± 1%, ApoE^−/−^ + Simvastatin 95% ± 1% *vs.* ApoE^−/−^ 60% ± 2%, *p* < 0.05). However, there was no significant difference in SNP induced and Ach induced (in presence of l-NAME) vasorelaxation of aortas from all groups (Figure 6B,C), showing that the vasorelaxing activities of xyloketal B is mediated by an endothelium-dependent NO generation mechanism. The doses of Ach and SNP causing half maximal response (EC50), expressed as pD_2_ (log_10_[EC50]) values, were calculated and are shown in Figure 6D. While the pD_2_ of Ach in xyl-B treated apoE^−/−^mice (−7.12 ± 0.04) tended to increase, it did not reach statistical significance as that in the Simvastatin group (−7.38 ± 0.04) in comparison to apoE^−/−^ mice (−6.94 ± 0.02). Also, there was no significant difference among all groups induced by SNP or Ach with l-NAME pretreatment. Taken together, these data suggested that xyloketal B could improve atherosclerotic endothelium dysfunction through an endothelium-dependent, NO-mediated mechanism.

### 2.5. Effect of Xyloketal B on Akt/eNOS Signaling in HUVECs

**Figure 7:**
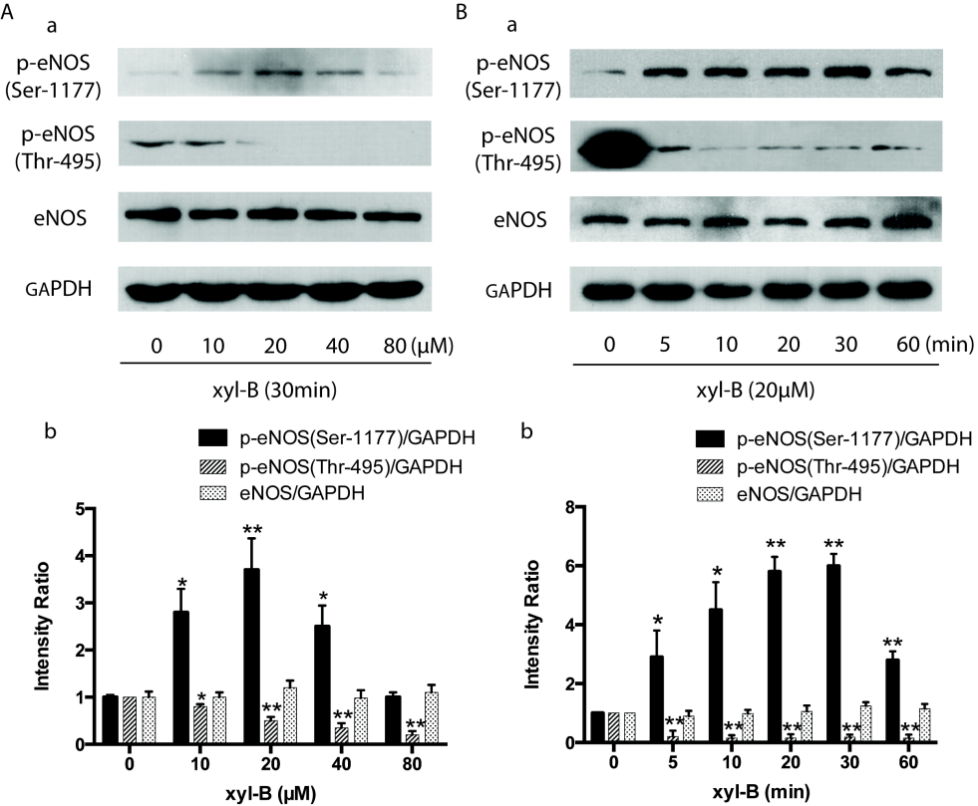

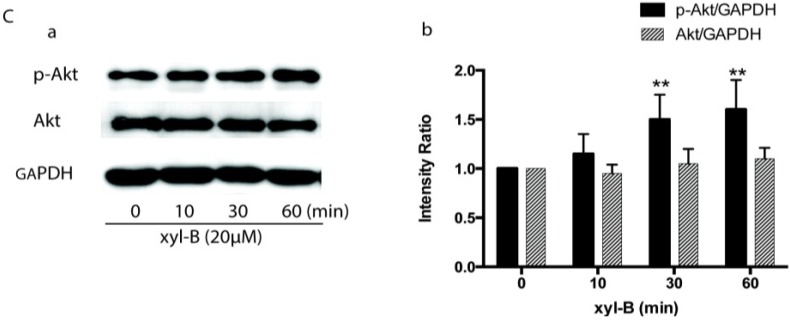
Effect of xyloketal B on phosphorylation of endothelial nitric oxide synthase (eNOS) and Akt in human umbilical vein endothelial cells (HUVECs). (**A**) Western blot analysis showing that xyloketal B affected eNOS phosphorylation in a concentration dependent manner. HUVECs were treated with xyloketal B at the indicated concentrations of 10–80 μM for 30 min. (**a**) Representative western blot showing the level of total eNOS expression and phosphorylation of eNOS at Ser-1177 and Thr-495. (**b**) The bar graph shows the densitometric analysis of the changes observed; (**B**) Western blot analysis showing that xyloketal B affected eNOS phosphorylation in a time-dependent manner. HUVECs were treated with 20 μM xyloketal B for the indicated time course (0, 5, 10, 20, 30, 60 min). (**a**) Representative Western blot showing the level of total eNOS expression and phosphorylation of eNOS at Ser-1177 and Thr-495. (**b**) The bar graph shows the densitometric analysis of the blots in (**a**); (**C**) Western blot analysis showing that xyloketal B affected Akt phosphorylation in a time dependent manner. HUVECs were treated with 20 μM xyloketal B for the indicated time course (0, 10, 30, 60 min). (**a**) Representative Western blot showing the level of total Akt expression and the phosphorylated Akt. (**b**) The bar graph shows the densitometric analysis of the blots in (**a**). All results of densitometric analysis of blots were normalized to 0 μM or 0 min. GAPDH was used as loading control. * *p* < 0.05, ** *p* < 0.01 *vs.* 0 μM or 0 min, *n* = 6.

To determine whether the protective effect of xyloketal B is due to its influence on the Akt/eNOS pathway, HUVECs were treated with different concentrations of xyloketal B and total eNOS protein expression and phosphorylation at Ser-1177 and Thr-495 were assessed by Western blot. As shown in Figure 7A,B, xyloketal B did not affect total protein expression of eNOS, however, it increased eNOS phosphorylation at Ser-1177 in a concentration and time-dependent manner. Incubation of HUVECs with 20 μM of xyloketal B for 20 min attained the maximal level of Ser-1177 phosphorylation (~six fold of control). In contrast, xyloketal B caused a dramatic inhibition of eNOS phosphorylation at Thr-495 only 5 min after xyloketal B treatment; 20 μM of xyloketal B also obtained the maximum inhibition (~90%). Up to this point we had demonstrated that xyloketal B protected endothelial dysfunction by affecting eNOS activity, we then further investigated upstream of eNOS phosphorylation. It has been reported that eNOS phosphorylation by PI3K/Akt pathway is required for efficient NO production [21,22], after phosphorylated at Ser-473 by PI3K, Akt in turn phosphorylates eNOS at Ser-1177 and thereby increases eNOS activity, and finally enhances NO production. In Figure 7C, we showed that 20 μM xyloketal B treatment could significantly increase Akt phosphorylation (~1.5 fold at 30 min) without altering its total protein expression in HUVECs. Taken together, these findings indicated that xyloketal B could increase eNOS activity by regulating phosphorylation of eNOS and Akt, which might also be responsible for the endothelial protective action of xyloketal B.

## 3. Experimental Section

### 3.1. Animals

Male apoE^−/−^ mice were switched to a high-fat diet (0.15% cholesterol and 21% fat) at 6 weeks of age and maintained on this diet for 16 weeks [23]. During this duration, xyloketal B (7, 14, 28 mg/kg/day respectively) or simvastatin (Merck, Kenilworth, NJ, USA, 10 mg/kg/day) [24] was administrated intraperitoneally or intragastrically respectively, and the same volume of soya bean oil was given as vehicle control. C57BL/6J mice fed a high-fat diet served as control. All animals were kept in certified specific pathogen-free facilities maintained around 24 °C with a 12-h light/dark cycle. All animal experiments were approved by the Animal Care and Use Committee of Sun Yat-sen University, and all animal care and experimental procedures strictly followed the Council for International Organizations of Medical Sciences (CIOMS) guidelines.

Our preliminary study on xyloketal B’s therapeutic effects on rat (~200 g) acute ischemia model yielded a therapeutic dose around 20 mg/kg/day [20]. We thus calculated the medium therapeutic dose of xyloketal B for mouse (20 g) using the formula D_A_ = K × D_B_, in which D_A_ represents the dose used in one kind of animal, D_B_ represents that in another kind and K is the conversion factor, which is 7 when it comes to the conversion between rat and mouse. Additionally, 20 mg/kg/day × 0.2 kg × 7/0.02 kg = 14 mg/kg/day, the initially working dosage was generated. After the dose of 14 mg/kg/day was confirmed to be effective, doses of 7 mg/kg/day and 28 mg/kg/day were thereby used as low dose and high dose, respectively.

### 3.2. En Face Analysis of Aortic Lesion

The extent of aortic atherosclerotic lesions in mice was examined by en face staining of aortas with oil red O as previously described [25]. Briefly, at the end of the treatment, mice were anesthetized with pentobarbital (60 mg/kg, i.p.). Aortas were removed 2 mm from the heart and excised from the aortic arch to just beyond the iliac bifurcation, cut longitudinally, fixed with 10% neutral buffered formalin (Beyotime Biotechnology, Haimen, China), stained with oil-red O (Beyotime Biotechnology, Haimen, China), and mounted on slides with the endothelium side up. Atherosclerotic plaques in full-length aorta, aortic arch, innominate artery (from the branching point to the Y-shaped bifurcation), thoracic aorta, and abdominal aorta were analyzed and quantified relative to the full-length lumen area, using the updated Image-Pro Plus program (Media Cybernetics, Silver Spring, MD, USA). Lesions were expressed as positive staining percentage for oil-red O of the total aortic area.

### 3.3. Histologic Analysis of Aortic Root Plaque

At the end of the treatment, mice were anesthetized with pentobarbital (60 mg/kg, i.p.). Heart was excised together with a short segment of aorta, stored in O.C.T. compound (SAKURA, Oakland, CA, USA), and quick-frozen on dry ice [26]. Cross-sections were cut and discarded until the three-valve cusps at the junction of the aorta to the heart became visible. Once this section was located, serial cross-sections (10 µm) were harvested, stained with 0.5% oil-red O solution and counter stained with Gill hematoxylin (Sigma Aldrich, St. Louis, MI, USA) for lesion evaluation. Total lesion area in the aortic root (mean of 3 sections per mouse) was measured blind to the identity of each section after oil-red O staining using the updated Image-Pro Plus program (Media Cybernetics, Silver Spring, MD, USA).

### 3.4. Detection of Serum Anti-OxLDL Autoantibodies

The serum titer of anti-oxidized low-density lipoprotein (oxLDL) autoantibodies was measured by an enzyme-linked immunosorbent assay (ELISA) with a Protein Detector kit (Kirkegaard Perry Labs, Gaithersburg, MD, USA) according to the manual provided. In brief, 96-well plate was coated with 100 μL of either OxLDL or native LDL and left overnight at 4 °C. After a washing step, the plate was then blocked with blocking solution at room temperature for 2 h. Mice serum samples were diluted 1:50 in blocking solution before addition to the wells. After additional overnight incubation at 4 °C, the serum samples were washed three times, and secondary antibody solution (1:200 alkaline phosphatase conjugated goat anti-mouse IgG in blocking solution) was added to each well and incubated at room temperature for 1 h. After extensive washing, 50 μL of substrate solution was dispensed into each well. The reaction was stopped after 30 min by the addition of 50 μL of stop solution to each well. Absorbance was detected at 405 nm in a Titertek ELISA reader (Bio-Tek Instruments, Highland Park Winooski, GA, USA). Results were expressed as absorbance at 405 nm.

### 3.5. Immunofluorescence Analysis of PECAM-1

Immunofluorescence staining was carried out on aortic sinus sections to measure the endothelial marker platelet endothelial cell adhesion molecule-1 (PECAM-1/CD31). Frozen sections of aortic sinus were fixed with acetone at −20 °C for 10 min and washed with PBS 3 times. Sections were incubated with 5% bovine serum albumin for 30 min at room temperature, followed by incubation with the primary antibody against PECAM-1 (1:100, BD Pharmingen, Franklin Lakes, NJ, USA) at 4 °C overnight, then reacted with Texas Red-conjugated secondary antibody (Beyotime Biotechnology, Haimen, China) for 30 min at 37 °C. All sections were analyzed using fluorescence microscope (ZEISS, Axio Imager Z1, Oberkochen, Germany, magnification 200×).

### 3.6. Transmission Electron Microscopy of Aortic Vessels

For transmission electron microscopic observation of ultrastructural changes in endothelial cells, aortic vessels were fixed with 3% glutaraldehyde and 4% paraformaldehyde in 0.1 M phosphate buffer. After dehydration with ethanol, the aortic vessels were embedded in Durcupan resin for ultra-thin sectioning and then were viewed under a transmission electron microscope (FEI TECNAI spirit G2, Hillsboro, OR, USA).

### 3.7. Measurements of Vasorelaxation of the Aortic Rings

At the end of the treatment, mice from different groups were anesthetized with pentobarbital (60 mg/kg, i.p.), thoracic aortas were dissected and immediately placed in oxygenated Krebs buffer containing (in mM) 137 NaCl; 5.4 KCl; 2.0 CaCl_2_; 1.1 MgCl_2_·6H_2_O; 0.4 NaH_2_PO_4_·2H_2_O; 5.6 Glucose·H_2_O; 11.9 NaHCO_3_, adherent connective tissues were removed softly. Aortas were cut into ~0.5 cm rings and equilibrated by means of 2 stainless steel hooks in an organ bath containing Krebs buffer at 37 °C, gassed with 95% O_2_/5% CO_2_. Isometric tension was measured with a force-displacement transducer connected to a wire myograph system (DMT, Aarhus, Denmark). The rings were set at a resting tension of 5 mN and equilibrated for 1 h, then contracted twice with KCl (60 mM). Phenylephrine (10^−6^ M), then, was added into the bath and caused in the vessels a sub-maximally contraction to 70% of the maximal response. After the plateau was reached, relaxant responses to the cumulative dose of acetylcholine (Ach, 10^−9^ to 3 × 10^−5^ M) were measured in the presence or absence of sodium nitroprusside (SNP, 10^−11^ to 10^−6^ M), which was applied to release nitric oxide. For endothelial independent relaxation measurement, arteries were incubated for 20 min with 30 μM NOS inhibitor l-NG-Nitroarginine Methyl Ester (l-NAME). Experiments were conducted in randomly chosen 4–6 pairs of rings from each group. Vasodilatation is expressed as a reduction percentage of the total vascular tone (myogenic tone plus Phe induced vasoconstriction).

### 3.8. Isolation and Culture of Endothelial Cells

Primary human umbilical vein endothelial cells (HUVECs) were cultured as we previously described [15,18]. Briefly, HUVECs were isolated from human umbilical veins from normal vaginal and caesarean section deliveries [27]. After 0.25% trypsin (Hyclone, Logan, UT, USA) digestion, the veins of the cells were washed out with PBS into M199 medium (Hyclone, USA) supplemented with 20% fetal bovine serum (FBS, Hyclone, UT, USA) to terminate the digestion. Cells were obtained after centrifugation for 5 min at 1000 rpm followed by soft resuspension and maintained on gelatin (Acumedia, Lansing, MI, USA) coated T25 flasks in M199 medium supplemented with 20% fetal bovine serum, 50 µg/mL endothelial cell growth supplement (ECGS, BD, Bedford, MA, USA), 50 ug/mL heparin (DINGGUO, Beijing, China). Cultures were maintained at 37 °C in a humidified 5% CO_2_ incubator. Primary culture medium was changed 8 h after seeding and cells were subcultured when reaching confluence of ~90% by use of 0.125% trypsin with 0.01% EDTA. HUVECs were identified by their typical cobblestone appearance and the presence of von Willebrand factor (factor VIII). HUVECs from 2 to 4 passages were used for the experiments.

### 3.9. Western Blot Analysis

HUVECs were treated with xyloketal B of the indicated concentrations and for the indicated duration of time. Western blot procedures were carried out as described previously [18]. Briefly, whole cell extract (40 µg) from each sample was analyzed with specific antibodies against eNOS (1:1000 dilution; BD Transduction Laboratories, Bedford, MA, USA), eNOS phosphoserine 1177 (1:1000 dilution), eNOS phosphothreonine 495 (1:1000 dilution), p-Akt (1:1000, Cell Signaling Technology, Danvers, MA, USA), Akt (1:1000, Cell Signaling Technology, Danvers, MA, USA). Incubation with polyclonal GAPDH antibody (1:2000 dilution; Jetway, China) was carried out as the loading control. The blots were detected on Kodak X-Omat film by enhanced chemiluminescence (Applygen Technologies, Beijing, China). Quantification of band intensity was carried out using Image J software (NIH, Bethesda, MD, USA).

### 3.10. Statistical Methods

All *in vitro* and *in vivo* data were from at least three independent experiments. Results were given as mean ± SEM. Statistical comparison among multiple groups was carried out by One-way ANOVA followed by LSD tests using Graphpad Prism 6. Statistical comparison between two groups was carried out using the Student’s *t-*test. *p* < 0.05 was considered statistically significant.

## 4. Discussion

The present study demonstrated that xyloketal B could reduce the aortic atherosclerotic lesion area in the entire aorta and aortic sinus in a concentration dependent manner, and thus alleviate the development of atherosclerotic plaques in apoE^−/−^ mice, suggesting it may have potential for treatment of atherosclerosis. Apolipoprotein E-deficient (ApoE^−/−^) mouse is the most popular animal model used in the study of atherosclerosis. Although ApoE^−/−^ mouse can spontaneously develop atherosclerotic lesions on a standard chow diet [28], Nakashima *et al*. have launched a research to compare the atherosclerotic lesions in mice fed with high-fat diet (Western diet) or chow diet, they concluded that high-fat diet resulted in much higher levels of cholesterol and much more serious atherosclerotic lesions, especially at the root of the aorta [23]. High-fat diet for 16 weeks is of great value for assessing whether a drug has a potential anti-atherogenic effect [29,30], because of the high number and large size of lesions throughout its aorta. Thus, it was used as an atherosclerotic animal model to investigate the protective effect of xyloketal B on atherosclerosis in our present study. It has been documented that high fat diet is associated with numerous cardiovascular diseases and metabolic diseases including obesity [31,32], hypertension [33], hyperglycemia [34], diabetes [35,36], and arrhythmias [37]; and high fat diet induced obesity has demonstrated sympathetic cardiac hyperinnervation and attenuated baroreflex function [37,38,39,40]. To specify the effects of xyloketal B on endothelium and oxidative stress, we employed the age-matched, high-fat diet fed C57BL/6J mice and vehicle-treated apoE^−/−^ mice as corresponding controls. In addition, we found that xyloketal B could improve the lipid metabolism disorder in atherosclerosis [20], although it did not affect the body weight of apoE^−/−^ mice fed with high-fat diet. In apoE^−/−^ mice, the beneficial effects of xyloketal B on the atherosclerotic lesion area were comparable to or stronger than simvastatin, a typical 3-hydroxy-3-methylglutaryl coenzyme A (HMG-CoA) reductase inhibitor. Our previous study revealed that xyloketal B could attenuate increasing blood pressure and myocardial remodeling in a two-kidney, two-clip stroke-prone hypertensive rat model [15,18]. Together with its safety, endothelial protecting effect, and strong anti-oxidant property, xyloketal B is a promising new drug candidate for the therapy of atherosclerosis. Our findings on the pharmacological effects of xyloketal B also suggest further *in vivo* studies on other cardiovascular disease models and may identify new therapeutic potential for xyloketal B.

Oxidative stress is crucially involved in the development of atherogenic endothelial dysfunction and atherosclerotic plaques [41]. The low-density lipoprotein (LDL) in the blood converts into oxLDL and then enters the vessel wall, which is the key step to initiate the vascular atherosclerotic process. The serum oxLDL antibodies level is thus taken as an indication of the oxidative status in the body and an important prediction of atherosclerosis [42]. OxLDL and anti-oxLDL antibodies have been detected in human plasma and in atherosclerotic lesions. Choi SH *et al.* showed that statin therapy significantly decreased oxLDL autoantibodies [43]. It was also reported that atorvastatin had an anti-atherogenic effect not by decreasing lipid levels, but by suppressing oxidation of LDL [44]. Consistent with these studies, we demonstrated that both simvastatin and xyloketal B could markedly reverse the increased oxidative stress throughout the body, which commonly occurs during atherogensis, providing for the first time the evidence that xyloketal B has a strong antioxidant action *in vivo*.

The disruption of endothelial integrity is an early key event during the pathogensis of atherosclerosis, and endothelial dysfunction was observed in apoE^−/−^ mice fed a high-cholesterol diet for 7 weeks [45]. Previously, we demonstrated that xyloketal B can protect against oxLDL-induced endothelial cell injury [15] in HUVECs, which is a well-recognized *in vitro* model for the development of endothelial protective agents. In the present study, immunofluorescence staining with antibody against PECAM-1 was performed in the aortic sinus sections. PECAM-1, which is abundantly expressed on the surfaces of many cells including monocytes, lymphocytes, platelets, and endothelial cells [7], is a widely used sensitive and specific marker of endothelium [46,47,48]. Immunofluorescence staining of PECAM-1 showed that aorta sinus sections from both xyloketal B and simvastatin treated apoE^−/−^ mice exhibited relative normal appearance of endothelium, in comparison to apoE^−/−^ mice, indicating that xyloketal B could maintain vascular endothelial integrity against atherosclerosis. The endothelial dysfunction during atherosclerosis is characterized by the impairment of endothelium-dependent vasorelaxation [49], which is ascribed to the reduced nitric oxide (NO) synthesis, excessive ROS production, over-generation of peroxynitrate radicals, and oxidative injury in the vascular endothelium. Our previous studies demonstrated that xyloketal B and other xyloketal derivatives can relax rat isolated thoracic aorta and promote endothelial NO production [16]. Here, we measured the arterial tension and found that xyloketal B could improve endothelium/NO dependent vasorelaxation activity in the atherosclerotic thoracic aorta. Notably, the protective effect of simvastatin in atherosclerotic formation shown in Figure 1 and Figure 2 is inconsistent with acetylcholine induced vasorelaxation in Figure 6. The different time points (16 weeks *vs.* 8 weeks) may be one explanation for the inconsistency of the endothelial protecting effects. The other possible explanation is that xyloketal B exhibits stronger anti-oxidant effects than simvastatin in atherosclerosis mice, which may contribute to the protection effects of xyloketal B on the endothelial integrity more than that on the vasorelaxation.

As our results above suggested, the protective effect of xyloketal B against atherosclerotic plaque formation was closely associated with its improvement on NO-mediated endothelial signaling. Endothelial NO synthase (eNOS) is the direct enzyme responsible for NO generation in endothelial cells [50,51] and also an attractive therapeutic drug target for cardiovascular diseases. The phosphorylation of eNOS at the Ser-1177 site has been well established as accelerating electron flux through the enzyme, thereby reducing the calcium requirement of eNOS and increasing NO production, making Ser-1177 a positive regulatory site [11,52]; conversely, the Thr-495 site serves as the negative regulatory site of eNOS activity by increasing calcium–calmodulin dependence of the enzyme [11,53]. Our previous study demonstrated that xyloketal B enhanced NO production in HUVECs in a time- and dose-dependent manner [15], which could be abolished by NOS blocker l-NAME. In this investigation, xyloketal B was found to increase eNOS activity by regulating phosphorylation of eNOS and Akt, which might also be responsible for the endothelial protective action of xyloketal B. Akt, PKC and calmodulin-dependent protein kinase are involved in the regulation of eNOS phosphorylation in endothelial cells [54]. The kinases involved in the process of phosphorylation of eNOS vary with the stimuli applied. It is known that the active kinase that phosphorylates eNOS at Ser-1177 is most probably Akt [21]. However, our study showed that 5 min treatment with 20 µM xyloketal B significantly increased the p-eNOS (Ser-1177), while 20 µM xyloketal B could not increase the p-Akt expression until 30 mins of incubation. It may suggest another kinase signaling pathway acting upstream of eNOS (Ser-1177). More detailed studies need to be performed for the xyloketal B-mediated phosphorylation of eNOS in the future.

## 5. Conclusions

Protection of vascular endothelium is well established as a therapeutic strategy for clinical prevention and treatment of atherosclerosis. Thus, clinical drugs and novel compounds with beneficial effects on endothelial dysfunction are of great promise for therapeutic inhibition of the progression of atherosclerotic plaque and other cardiovascular diseases.

In the present study, we demonstrated that xyloketal B could attenuate the atherosclerotic lesion area in atherosclerotic apoE^−/−^mice. The maintenance on endothelial integrity, improvement on NO-generated endothelial relaxation and decrease of levels of oxidative stress are responsible for its anti-atherogenic effects. Another of our ongoing studies has found that xyloketal B could partly decrease the serum LDL level [20], suggesting that this marine drug candidate may have a beneficial influence on the different mechanisms of atherosclerosis. The regulation of phosphorylation of the Akt/eNOS pathway may be ascribed to the protective effects of xyloketal B on endothelial cells and will need further investigation. Given that xyloketal B is very safe and has a low molecular weight and high liposolubility, it could be absorbed quickly when administered intragastricly and therefore has great potential for the development of new drugs against cardiovascular diseases related to endothelial dysfunction.
